# Sustainable production of bacterioruberin carotenoid and its derivatives from *Arthrobacter agilis* NP20 on whey-based medium: optimization and product characterization

**DOI:** 10.1186/s40643-023-00662-3

**Published:** 2023-07-24

**Authors:** Nehad Noby, Sherine N. Khattab, Nadia A. Soliman

**Affiliations:** 1 Department of Biotechnology, Institute of Graduate Studies and Research, Alexandria University, Alexandria, 21526 Egypt; 2 Department of Chemistry, Faculty of Science, Alexandria University, Alexandria, 21321 Egypt; 3 Bioprocess Development Department, Genetic Engineering & Biotechnology Research Institute (GEBRI), City of Scientific Research & Technological Applications, (SRTA-City), New Borg Elarab, Alexandria, Egypt

**Keywords:** *Arthrobacter agilis*, Cost-effective medium, Glycosylated bacterioruberin derivatives, Radical scavenging activity, Response surface methodology, Valorization of cheese whey

## Abstract

Bacterioruberin and its rare glycosylated derivatives are produced by *Arthrobacter agilis* as an adaptation strategy to low temperature conditions. The high antioxidant properties of bacterioruberin held great promise for different future applications like the pharmaceutical and food industries. Microbial production of bacterioruberin via a cost-effective medium will help increase its commercial availability and industrial use. The presented study aims to optimize the production of the rare C_50_ carotenoid bacterioruberin and its derivatives from the psychotrophic bacteria *Arthrobacter agilis* NP20 strain on a whey-based medium as a cost effective and readily available nutritious substrate. The aim of the study is extended to assess the efficiency of whey treatment in terms of estimating total nitrogen content in treated and untreated whey samples. The significance of medium ingredients on process outcome was first tested individually; then the most promising factors were further optimized using Box Behnken design (BBD). The produced carotenoids were characterized using UV–visible spectroscopy, FTIR spectroscopy, HPLC–DAD chromatography and HPLC-APCI-MS spectrometry. The maximum pigment yield (5.13 mg/L) was achieved after a 72-h incubation period on a core medium composed of 96% sweet whey supplemented with 0.46% MgSO_4_ & 0.5% yeast extract and inoculated with 6% (v/v) of a 24 h pre-culture (10^9^ CFU/mL). The cost of the formulated medium was 1.58 $/L compared with 30.1 $/L of Bacto marine broth medium. The extracted carotenoids were identified as bacterioruberin, bis-anhydrobacteriouberin, mono anhydrobacterioruberin, and glycosylated bacterioruberin. The presented work illustrates the possibility of producing bacterioruberin carotenoid from *Arthrobacter agilis* through a cost-effective and eco-friendly approach using cheese whey-based medium.

**Graphical Abstract:**

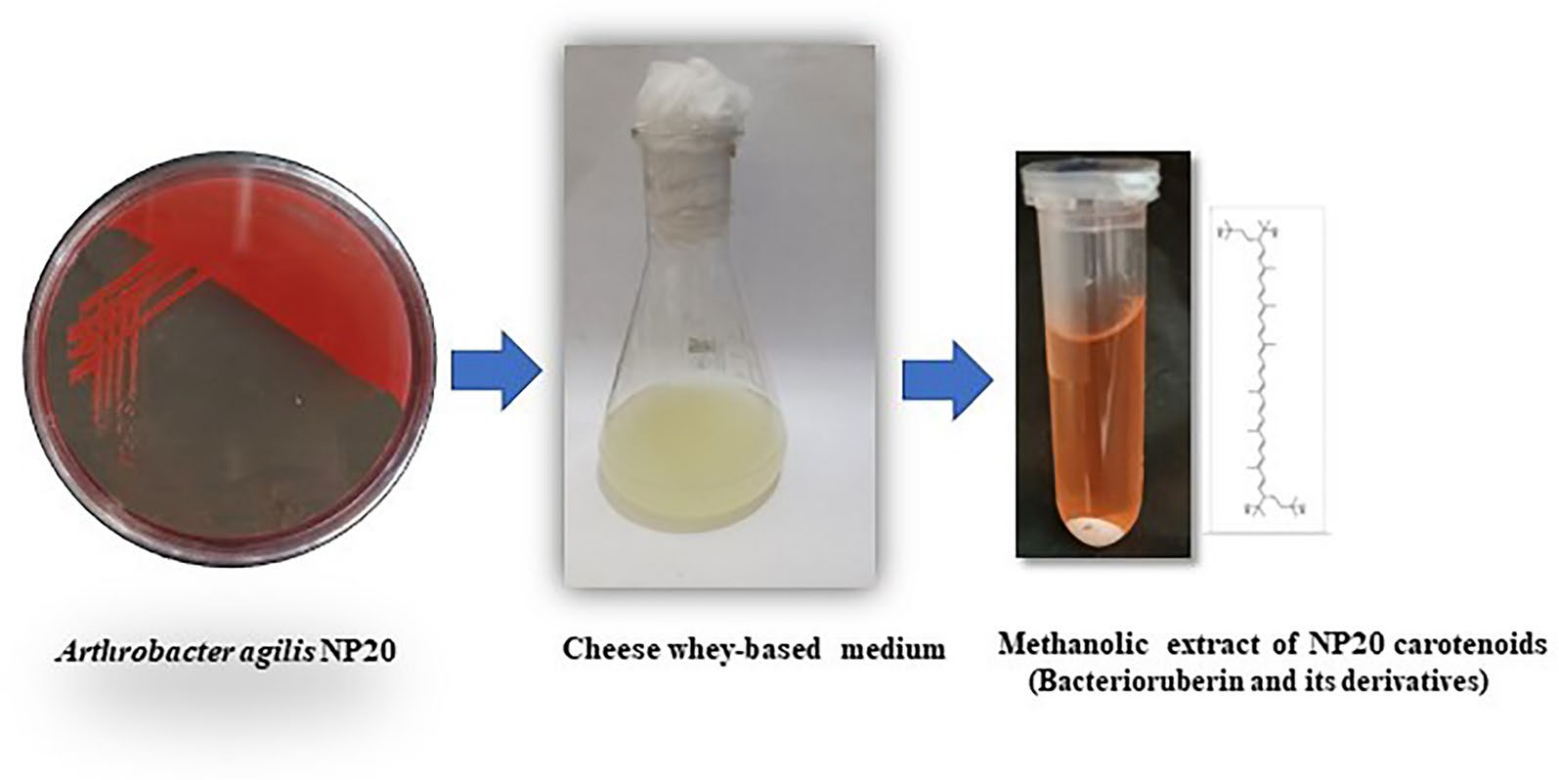

**Supplementary Information:**

The online version contains supplementary material available at 10.1186/s40643-023-00662-3.

## Introduction

Recently, environmental and health-related worries about the hazards of synthetic colorants have sparked interest in finding natural alternative dyes, especially for food and cosmetic applications (Celedón and Díaz 2021). In response to this, an upsurge in the demand for natural dyes has been noticed in the past few years to replace synthetic ones (Yusuf et al. 2017). Although microorganisms produce pigments in multiple elegant shades with different bioactivities like anticancer, antioxidant antimicrobial, and UV-protective properties (Silva et al. 2014; Agarwal et al. 2023), microbial-based pigments have not been widely used in several industries due to the lack of commercial availability. Successful implementation of microbial cell factories for the sustainable production of natural carotenoids can be achieved by making them economically competitive with synthetic ones. The cost of the nutritional medium used to grow the microorganisms is one of the main obstacles that hinders large-scale production of microbial-based pigments (Aman Mohammadi et al. 2022). To overcome this obstacle, many efforts have been directed towards utilizing low-cost substrates to provide the necessary elements for microbial growth. Biomasses produced from the processing of agricultural and food products are highly rich in carbohydrates, proteins, vitamins, and minerals. However, most of these wastes are not optimally used and cause environmental pollution (Jin et al. 2018). Recycling these wastes into value-added products like bio pigments is a sustainable approach to satisfying the increasing needs of bio-colors and would help strengthen the circular economy concept (Usmani et al. 2020). Many agri-food by-products such as fruit pomace, seeds, peels, corn steep liquor, molasses, whey, bran, etc. have been utilized as possible fermentable substrates to produce bio-pigments using bacteria, yeast, fungi, and algae. For instance, corn steep liquor and preboiled rice water were used as food waste substrates to produce β-carotene using *Sporidiobolus pararoseus* (Valduga et al. 2014). Prodigiosin was produced from *Serratia marcescens* using rice bran as a substrate (Arivizhivendhan et al. 2018).

Bacterioruberin pigment is among the rare carotenoids produced exclusively by a limited number of microorganisms. It’s a bright red pigment, classified as C50 carotenoids, formed from an isoprenoid chain with 13 conjugated C = C units and four functional OH-groups arising terminally (Dummer et al. 2011; Yang et al. 2015). The long conjugated double bond system of bacterioruberin has contributed to its superior antioxidant properties compared to β-carotene, which comprises only nine conjugated double bonds (Maia et al. 2021). The high antioxidant capacity of bacterioruberin makes it a promising ingredient in pharmaceutical (Hou and Cui 2018; Rodrigo-Baños et al. 2015) and cosmeceutical products like anti-ageing and sunscreen formulations (Nichols and Katiyar 2010; Galasso et al. 2017).

Bacterioruberin was reported to play an adaptive role in producing microorganisms. It is produced in a stress-dependent manner under harsh conditions such as high salt content or low temperature conditions (Sutthiwong et al. 2014). For instance, Bacterioruberin carotenoid and its derivatives are the main carotenoids of Haloarchaea that grow optimally in hypersaline environments with an approximate salt concentration of 25–30% (w/v) (Giani et al. 2019). It was proven that bacterioruberin helps this group of archaea thrive under hypersaline conditions by acting as a rivet, allowing the permeability of oxygen and other molecules and preventing water passage (Abbes et al. 2013). On the other hand, bacterioruberin is produced in a temperature-dependent manner in two psychrophilic species of the Arthrobacter genus, *Arthrobacter agilis* and A. *bussei,* (Flegler et al. 2020; Fong et al. 2001a). Recent studies have confirmed the role of bacterioruberin and its glycosylated derivatives in improving membrane fluidity at low temperatures and reducing the freezing–thawing effect on bacterial membranes (Flegler and Lipski 2021, 2022a).

It is noteworthy to highlight that the glycosylated derivatives of bacterioruberin are specifically produced in *Arthrobacter* sp. to cope up with its role in cold adaptation, since carotenoid glycosides are found to cluster in rigid patches, increasing the local rigidity of the membrane to resist the effect of low temperature (Chen et al. 2021; Mohamed et al. 2005; Várkonyi et al. 2002). Glycosylated carotenoids are of high industrial value since glycosylation enhances the solubility, photo-stability, bioavailability, and biological activities of the produced carotenoids (Chen et al. 2021). The quality of carotenoids produced by *Arthrobacter* sp. makes it a favorable source of bacterioruberin carotenoids compared to the Haloarchaea group.

In this context, the introduced research focuses on producing the rare C50 bacterioruberin carotenoid and its derivatives via a cost-effective and eco-friendly way from *Arthrobacter agilis* using a whey-based medium. The medium composition was optimized via the response surface methodology (RSM) approach. The work is extended to characterize the produced pigments and test their antioxidant capacity. To the best of our knowledge, the presented research is the first to handle bacterioruberin production from *Arthrobacter agilis* using a waste-based medium.

## Material and methods

### Growth media

PY medium (10 g L^−1^ peptone, 5 g L^−1^ yeast extract, 5 g L^−1^ NaCl) was used in isolation step. PA is the solidified form of PY medium using 1.5% agar. Cheese-whey-based medium (96% sweet-whey, 0.46% MgSO4, 0.5% yeast extract) was used as the optimized medium for carotenoid production.

Sweet whey was kindly supplemented from the faculty of Agriculture, Alexandria University.

### Isolation, identification of carotenoid producing strain

Isolation step was carried out from air and soil samples. Isolation from air was carried out through settle plate technique, in which sterile PA plates were exposed to air for 15 min then the plates were incubated at 25 °C. Among the grown colored colonies, a pink pigmented one was picked up, purified, and stored at − 20 °C in 15% glycerol.

The selected strain was examined morphologically and microscopically. In addition, the strain was subjected to molecular identification through amplifying 16S*rRNA* encoding gene according to the previously reported method (Eden et al. 1991). Genomic DNA was extracted using quick DNA miniprep kit (D3024, ZYMO), according to manufacturer’s instructions. The whole length of 16S*rRNA* gene was amplified through polymerase chain reaction (PCR) using the universal primers F27, R1492. The sequence of the purified PCR product was determined via the automated fluorescent DNA sequencer. The obtained nucleotide sequence was analyzed using BLAST n tool offered by NCBI, and the phylogenetic analysis was carried out using MEGA X software to determine the taxonomic affiliation of the isolate.

### Pigment extraction and quantification

Fresh activated colonies were picked up for inoculating 20 mL PY medium, the culture was incubated for 24 h at 25 °C. An inoculum of 5% of the grown preculture used to inoculate 100 mL fresh PY medium sterilized in 250 mL flask and incubated at 25 °C for 72 h. At the end of the incubation period, cells were harvested by centrifugation at 4 °C for 10 min at 10,000 rpm. Cell pellets were suspended and soaked in methanol for 2 h until complete bleaching of the cells. The extract was centrifuged at 4 °C for 10 min at 10,000 rpm for removing residual cells, wrapped with aluminum foil for light protection, and then stored in − 20 °C.

The extracted pigment was scanned in a wavelength region of 200–800 nm. Total carotenoid concentration in the methanolic extract was determined at the obtained maximum wavelength (λ_490_) through applying an average extension coefficient (Liaaen-Jensen and Jensen 1971).

### Correlation of growth pattern and pigment synthesis of *Arthrobacter agilis* NP20

To determine maximum production time of the pigment, pigment synthesis was correlated to dry weight over 5 days. The isolate was allowed to grow in PY medium as illustrated previously. At constant time intervals, a suitable culture volume was harvested by centrifugation, pigment was extracted and quantified from the obtained cell pellets as mentioned before, and finally the remaining cell mass was dried at 70 °C until reaching a constant weight.

### Optimization of C50 carotenoid production on whey-based medium

The capability of the isolate for pigment production was tested on whey-based medium. The basal medium (nominated as Medium 1) is composed of 50% sweet-whey, 0.5% yeast extract and inoculated with 5% of a pre-culture of the same composition. One factor at a time approach was applied to assess the significance of MgSO_4_, NH4Cl, and KH_2_PO_4_ on process outcome. A concentration of 1% of MgSO_4_, NH4Cl, and KH_2_PO_4_ was added individually to medium 1, constituting medium 2, 3, and 4, respectively. In addition, the significance of omitting yeast extract from medium composition was also assessed (Medium 5). In each trial, pigment concentration and cell dry weight were estimated to evaluate the impact of each variable. All trials were carried out in triplicates at 20 °C and 200 rpm for 72 h incubation time. Significant difference was considered at *P*-value ≤ 0.05 according to the overlapping rule of standard error bars reported by Cuming et al. (Cumming et al. 2007).

The most significant variables were subjected to further optimization step using RSM to estimate their optimal values besides determining the significant interactions between them. In this context, the three selected variables MgSO4% (X1), cheese-whey % (v/v) (X2), inoculum size % (v/v) (X3) were considered to BBD analysis each variable was tested at low (− 1) middle (0) and high level (+ 1) in terms of coded values.

Experimental trials were carried out in 250 mL Erlenmeyer flask with a working volume of 30 mL. All trials were incubated at 20 °C, 200 rpm, for 72 h.

RSM determines all possible interactions of the involved independent variables via second-order polynomial equation. The three independent variables of this study are presented by the following equation:

1 $$ Y \, = b_0 + b_1 \left( {X_1 } \right) \, + b_2 \left( {X_2 } \right) \, + b_3 \left( {X_3 } \right) \, + b_{12} \left( {X_1 X_2 } \right) \quad\, + b_{13} \left( {X_1 X_3 } \right) \, + b_{23} \left( {X_2 X_3 } \right) \, + b_{11} \left( {X_1 } \right)^2 + b_{22} \left( {X_2 } \right)^2 + b_{33} \left( {X_3 } \right)^2 $$

where, Y is the predicted response, β0 is constant, β1, β2, and β3 are linear coefficients, β12, β13, and β23 are cross product coefficients, and β11, β22, and β33 are quadratic coefficients.

#### Data analysis

The JMP tool was used to run multiple linear regressions on the pigment production data to estimate *t*-values, *p*-values, and confidence levels. The *p*-values were reported as percentages. The significance level was assessed using the *Student t test* (*p*-value). The *t test* for every specific impact allows one to determine how likely it is that the observed effect was discovered accidentally. If the probability of the test variable is low enough, it will be acceptable. The *p*-value is represented as a percentage by the confidence level. The STATISTI CA 7.0 programme created three-dimensional and contour plots to show the simultaneous effects of the three most significant independent factors on each response.

#### Model verification

The optimum conditions found through optimization trials were put to the test empirically and contrasted with the results of the model. To prove the accuracy of the model, the model % error was calculated from the following formula:

$$ {\text{Model percentage error }} = \, \left\{ {\left( {Y{\text{ Experiment}} - Y{\text{ calculated}}} \right) \, / \, Y{\text{ Calculated}}} \right\} \, \times {1}00 $$

### Determination of nitrogen content in the utilized whey before and after treatment.

The total nitrogen content in treated and untreated whey samples was assessed using Kjeldahl method with some modifications. Briefly, 20 mL of H_2_SO_4_ and a Kjeldahl tablet was added to 2 mL of both treated and untreated whey samples, separately. The reaction content was burned to 450 °C until it changed into a clear blue green color. After cooling, it was given a quick stir before receiving 200 mL of distilled water and 80 mL of a potassium sulfide-containing NaOH solution. It was associated with the distillation process in this way. On the opposite side of the apparatus, 25 mL of a 3% boric acid solution with Congo red as an indicator were dropped. The nitrogen ratio was computed after the distillation operation was completed and titrated using 0.1 N NaOH solution.

### Chromatographic analysis for carotenoid identification

The extracted carotenoids were characterized by UV–visible spectroscopy, FTIR spectroscopy, HPLC–DAD chromatography and HPLC-APCI-MS spectrometry.

In FTIR analysis, the methanolic extract of the carotenoids was applied to salt cuvette and analyzed by FTIR spectroscopy (Perkin Elmer, Germany). The device spectral range was adjusted between 450 and 4000 cm^−1^.

For HPLC chromatography, a volume of 100 µL of the prefiltered pigment extract was injected into C_18_ reversed phase column (250 mm × 4.5 mm, 5 µm, Phenomenex, USA). Separation of carotenoids was achieved using two solvent systems, solvent A (H_2_O: MeOH (1:9 v/v)), and solvent B (EtOAc: MeOH (1:9 v/v)). The elution was carried out gradually; where solvent B concentration was raised from 0 to 100% over 30 min. Eluted peaks were detected using PDA detector in the range 200–600 nm, while the chromatogram was built up at 490 nm. In addition, pigment composition was further investigated via mass spectrometry, the mass spectrum was determined in the range of 200–3000 m/z using the positive ionization mode. Carotenoids identification was determined through comparing the fraction retention time and the characteristics of both UV and mass spectra.

### Antioxidant effect

The antioxidant power of *Arthrobacter agilis* NP20 carotenoids was assessed through free radical scavenging activity (RSA) using DPPH, and the RSA efficiency was compared to β-carotene standard. The assay was applied according to the method previously mentioned (Jiménez-Escrig et al. 2000). Briefly, different concentration of the extracted carotenoids (1.5, 5 and 7 µg) and a concentration of 50 µg of β-carotene standard were tested for their antioxidant efficacy. The samples were diluted to 350 µL with methanol and mixed with 1 mL of 100 µM DPPH. The negative control test was prepared by adding 350 µL of absolute methanol to 1 mL of DPPH. The reactions were incubated at room temperature and the discoloration of DPPH was monitored at wavelength 580 nm along 40 min.

The RSA scavenging efficiency was calculated according to Eq. 2:

2 $$\mathrm{RSA\%}=1-\left(\frac{\mathrm{Sample\,Absorbance }}{\mathrm{Negative\,Control\,Absorbance }}\right)x100$$

## Results

### Isolation and identification of the red pigmented isolate

**Fig. 1:**
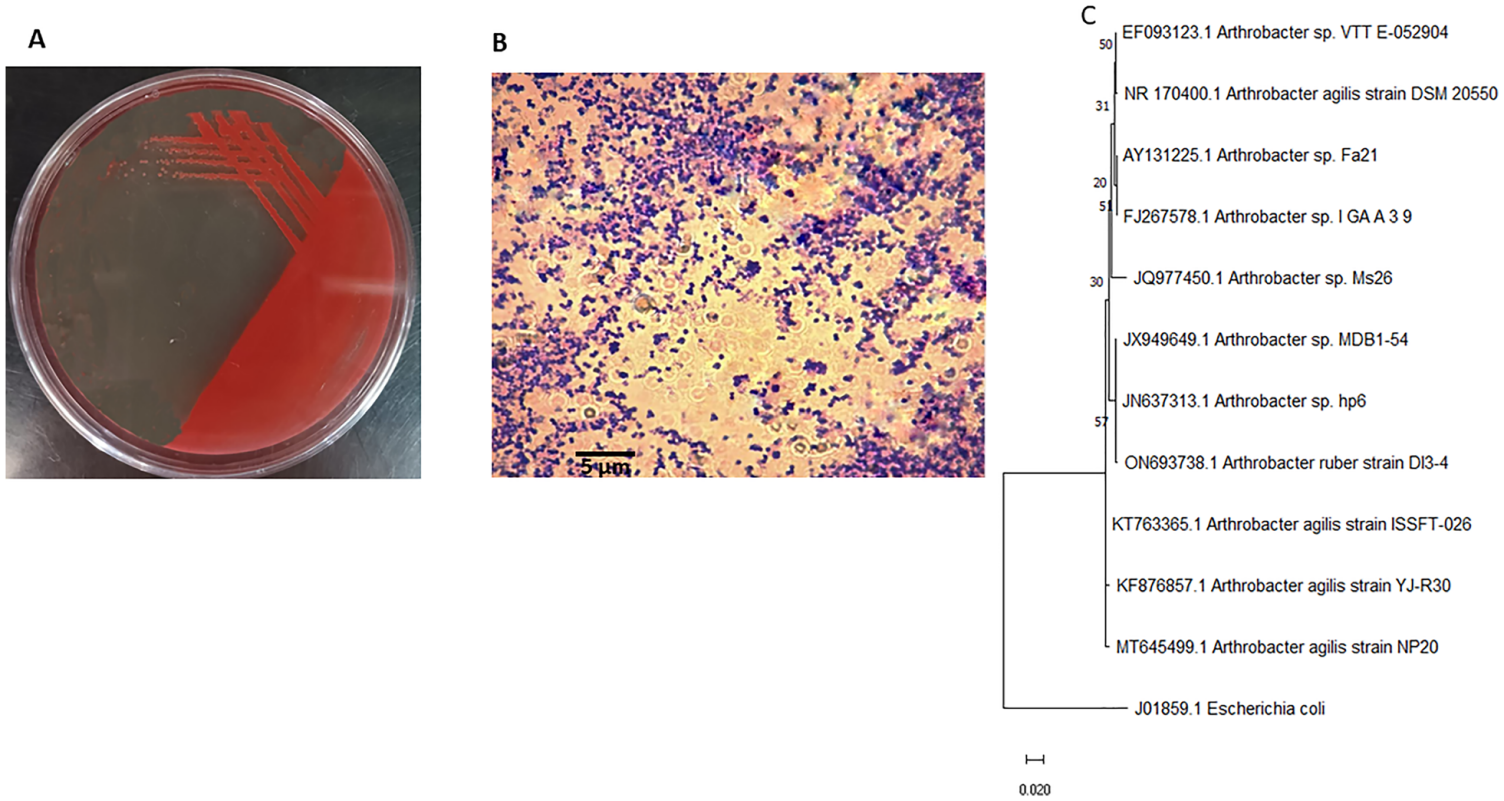
Identification of *Arthrobacter agilis* strain NP20 **A** Phenotypic characterization of colony morphology showing Pink pigmented colonies grown on PY medium **B** cell morphology under the microscope showing cocci-shaped cells **C** Phylogenetic analysis using Maximum likelihood method for estimating the relatedness of Arthrobacter agilis NP20 and closely related bacteria. The accession number is displayed by each genus. Bootstrap values are displayed as a percentage of 500 replicates. Escherichia coli was used as an outgroup

The isolation step has resulted in 3 carotenoid producing isolates of different hue colors. However, due to the scarcity of naturally synthesized red carotenoids, red pigmented isolate was selected for further study. On PA medium, colonies appeared smooth, rounded, with reddish pink color with noticeable color darkness after storage at 4 °C (Fig. 1A). Cells of *Arthrobacter agilis* appeared cocci under the microscope (Fig. 1B).

Analysis of 16S*rRNA* sequence revealed that the isolate is affiliated to *Arthrobacter agilis* (Fig. 1C). The bacterium was nominated as *Arthrobacter agilis* NP20 and deposited in GenBank under accession number (MT645499).

### Production and identification of red carotenoid

**Fig. 2:**
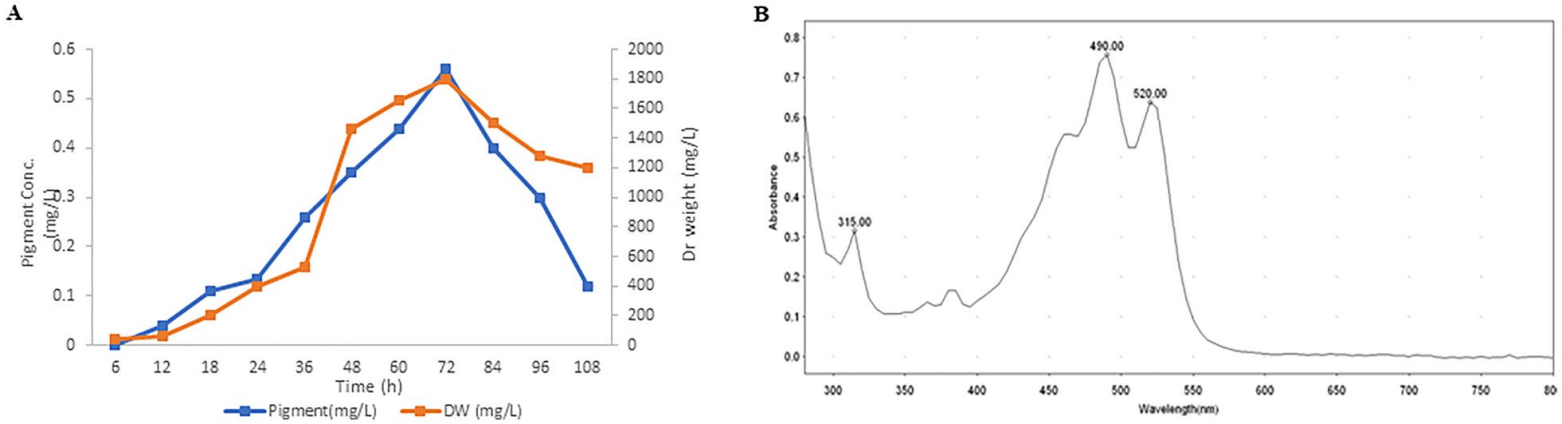
Production and extraction of red pigment carotenoid from *Arthrobacter agilis* NP20. **A** Biomass and pigment accumulation pattern of NP20 strain cultivated on whey-based medium along 5 days of incubation **B** Visible spectrum of the extracted pigment showing the typical (three fingered) maxima of red carotenoids with maximum absorbance around 500 nm

The UV/VIS spectrum of pigment methanolic extract showed a characteristic 3 fingered maxima distinctive to C50 carotenoids with maximum absorption at 490 nm (Fig. 2B).

**Fig. 3:**
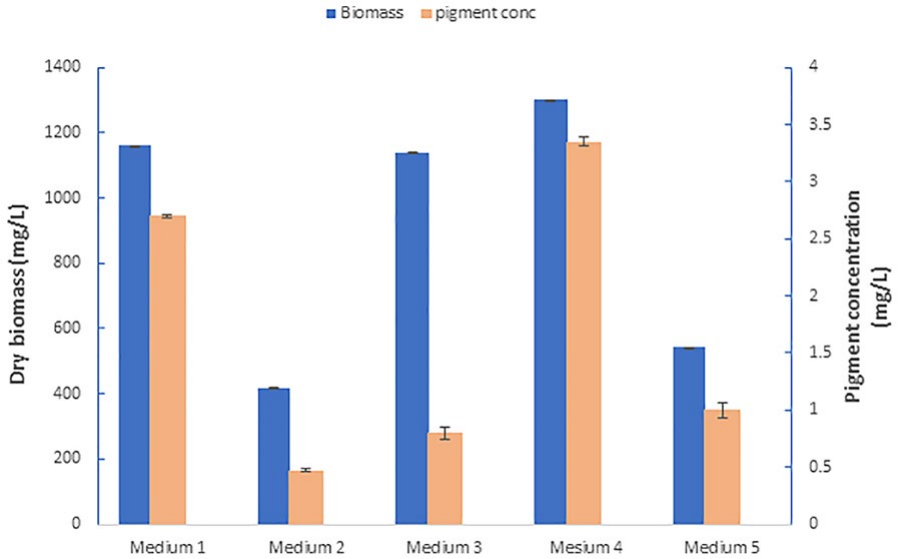
Effect of changing medium ingredients on pigment and biomass yield. **Medium 1** consists of 50% whey and 0.5% yeast extract. **Medium 2**: 1% NH4Cl added to medium1, **Medium 3**: 1% KH2PO4 added to medium1, **Medium 4**: 1%MgSO4 added to medium 1, **Medium 5**: 50% Sweet. All trials were carried out in triplicates at 20 °C. The significant difference was considered according to the overlapping rule at P-value ≤ 0.05. (Cuming et al. 2007)

To estimate maximum production time of NP20 carotenoids, the production pattern was related to the strain growth curve in terms of biomass accumulation (Fig. 3A). As illustrated in Fig. 3A, pigment accumulation pattern typically follows the growth pattern of the isolate, where pigment production increased gradually from the onset of log phase and reached its maximum value at the end of the stationary phase. After 72 h, the strain entered a decline phase which displayed a noticeable drop in pigment content due to pigment leakage outside dead cells.

### Optimization of *Arthrobacter agilis* NP20 pigment production using BBD

Among the added supplements tested initially (Fig. 3), only medium 2 showed a significant increase in both pigment and biomass accumulation. The omission of 0.5% of yeast extract from medium composition (medium 5) severely affected the process outcome (Fig. 3). However, higher concentrations of yeast extract negatively affected pigment accumulation (data not shown).

Based on the obtained results, a fixed concentration of yeast extract was used, while the optimal concentration of both MgSO4% (X1) and whey % (X2) along with the inoculum size % (X3) were further tested using BBD.

The variables were screened at three encoded levels, − 1, 0, 1 in a collective matrix of 15 trials. Table1 shows the designed matrix, real variables levels and the response of each trial in terms of pigment concentration (mg/L) quantified at 490 nm.

**Table 1:** Matrix for BBD with real and coded values of three independent variables along with the resulted responses of pigment production in term (mg/L)

	Variables			Pigment conc. (mg/L)		
Trial	X1	X2	X3	Experimental	Predicted	Residual*
1	0	0	0	3.44	3.38	0.06
2	0	0	0	3.37	3.38	− 0.01
3	0	-1	1	2.75	2.50125	0.24875
4	0	1	-1	4.2	4.44875	− 0.24875
5	− 1	− 1	0	3.19	3.54125	− 0.35125
6	0	0	0	3.33	3.38	− 0.05
7	1	0	− 1	3.8	3.9025	− 0.1025
8	0	1	1	3.7	4.19625	− 0.49625
9	0	− 1	− 1	2.8	2.30375	0.49625
10	− 1	0	1	4.24	4.1375	0.1025
11	− 1	0	− 1	3.34	3.485	− 0.145
12	1	0	1	3.34	3.195	0.145
13	1	1	0	5.55	5.19875	0.35125
14	1	− 1	0	2.63	3.02375	− 0.39375
15	− 1	1	0	5.6	5.20625	0.39375

Variables	Coded level and actual level		
	− 1	0	1
X1: % MgSO4 (w/v)	0.1	0.3	0.5
X2: % Whey (v/v)	40	70	100
X3: %Inoculum size (v/v)*	5	10	15

*One mL of mother culture contains 4 × 10^9^ CFU

*Residual = actual value of studied response–predicted value of studied response, a negative residual means that the predicted value is high and a positive residual means that the predicted is low

**Fig. 4:**
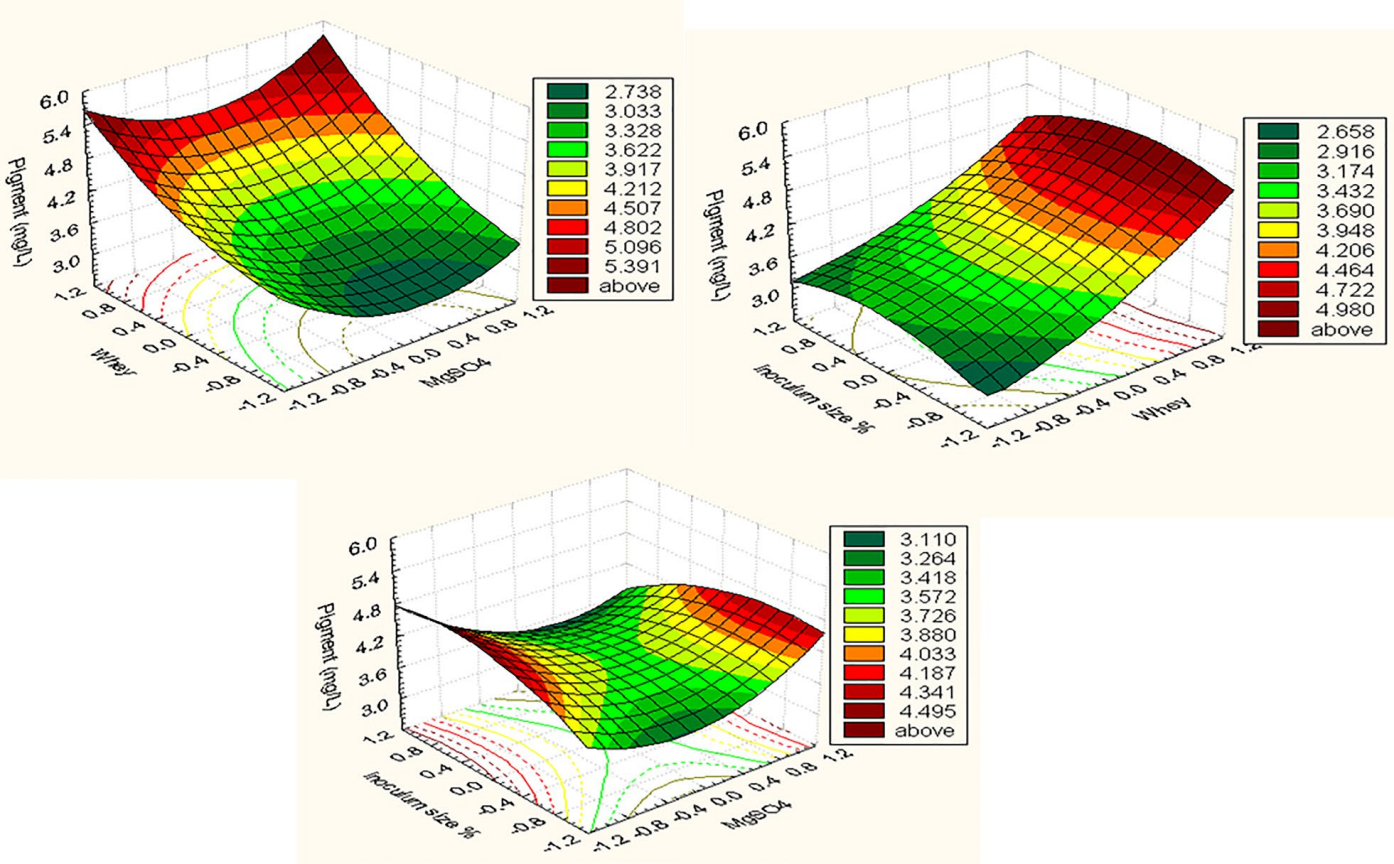
Three-dimensional surface and contour plots showing the relationships between the tested variables and the pigment production by *Arthrobacter agilis* NP20

Model significance was inferred from *P*-value (0.056) and *F* value (4.1) displayed by ANOVA analysis (Table 2). The small regression value (*P* = 0.056) proves the accuracy of the model in elucidating the relationship between the response and the involved independent variables. The value of *R*^*2*^ was 0.8897 indicating a high degree of association between the predicted and experimental values. Actual by predicted plot for the measured response (pigment conc. (mg/L)) is shown in (Additional file 1: Fig. S1), where a regression line is to minimize the sum of residuals. The multiple linear regression models describe the relationship between the pigment production level and three studied variables namely, MgSO4, whey % and the inoculum size %. Surface plots (Fig. 4) show the variables interaction and contour analyses in plots were calculated to detect the centre point which gives maximum production.

**Table 2:** Statistical analysis of BBD showing *coefficients, t –*and *p-values* for significant variables affecting on pigment production by *Arhrobacter agilis* strain NP20

Term	Coefficients	Standard Error	t Stat	P-value	Upper 95.0%
Intercept	3.38	0.287793	11.7445391	7.87E-05	4.119796
X1	− 10.13125	0.176237	− 10.744737043	0.489932	0.321781
X2	0.96	0.176237	5.447219513	0.002832	1.413031
X3	− 10.01375	0.176237	− 10.078020071	0.940838	0.439281
X1X2	0.1275	0.249236	0.511562653	0.630741	0.768182
X1X3	− 10.34	0.249236	− 11.364167074	0.230711	0.300682
X2X3	− 10.1125	0.249236	− 10.451378811	0.67062	0.528182
X1X1	0.59	0.259413	2.274362073	0.072044	1.256843
X2X2	0.2725	0.259413	1.05044689	0.341609	0.939343
X3X3	− 10.29	0.259413	− 11.117906782	0.314422	0.376843

ANOVA	*df*	SS	MS	*F*	Prob > *F*
Model	9	10.028598	1.11429	4.4845	0.0565
Error	5	1.242375	0.24847		
C. Total	14	11.270973			
RSquare	0.889772				
RSquare Adj	0.691362				
Root Mean Square Error	0.498473				
Mean of Response	3.685333				
Observations	15

The obtained response results were integrated in a second-order polynomial equation to determine the exact value of each variable and to predict the maximum pigment concentration. The built equation (Eq. 3) includes all possible forms of interactions:

3 $$ {\text{Y}}_{{\text{pigment yield mg}}/{\text{L}}} = { 3}.{38 } - \quad 0.{\text{131X1}} + \, 0.{\text{96 X2 }} - \, 0.0{\text{137 X3 }} + \, 0.{\text{1275 X1X2 }} - \quad 0.{\text{34 X1X3 }} - \, 0.{\text{112 X2X3 }} + \quad 0.{\text{59 X1X1 }} + \, 0.{\text{272 X2X2 }} - \, 0.{\text{29 X3X3}} $$

**Fig. 5:**
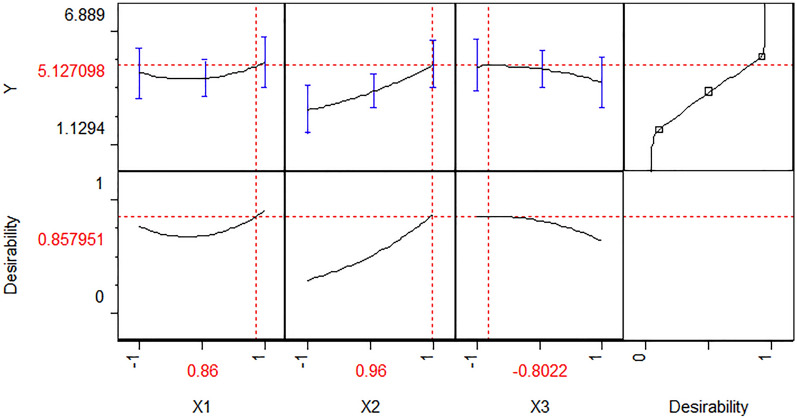
JMP Desirability prediction profile showing the predicted optimal coded levels (0.86, 0.96 and − 0.80) of studied three variables MgSO4, Whey and inoculum size, respectively, to maximize the pigment production of *Arthrobacter agilis* NP20

Differentiating the polynomial equation revealed optimal variable values to be (*X*1 = 0.86), (*X*2 = 0.96), and (*X*3 = − 0.80) in terms of coded values to yield 5.13 mg/L as maximum predicted pigment yield (Fig. 5).

A verification experiment was run under the previously obtained anticipated optimal condition to assess the quadratic polynomial's accuracy. The percent error formula was determined as the absolute value of the difference of the measured value and the actual value divided by the actual value and multiplied by 100.

The measured pigment level of NP20 strain was 6.01 mg/L; this means that the calculated model percentage error [(6.01–5.13)/5.13]*100 was ~ 17%.

### Determination of nitrogen content in whey before and after treatment

According to Kjeldahl method, the total nitrogen content in the untreated sample recorded 0.56% while the value reduced to 0.14% after microbial treatment using the optimized conditions mentioned above.

### Analysis and characterization of the extracted carotenoids

**Fig. 6:**
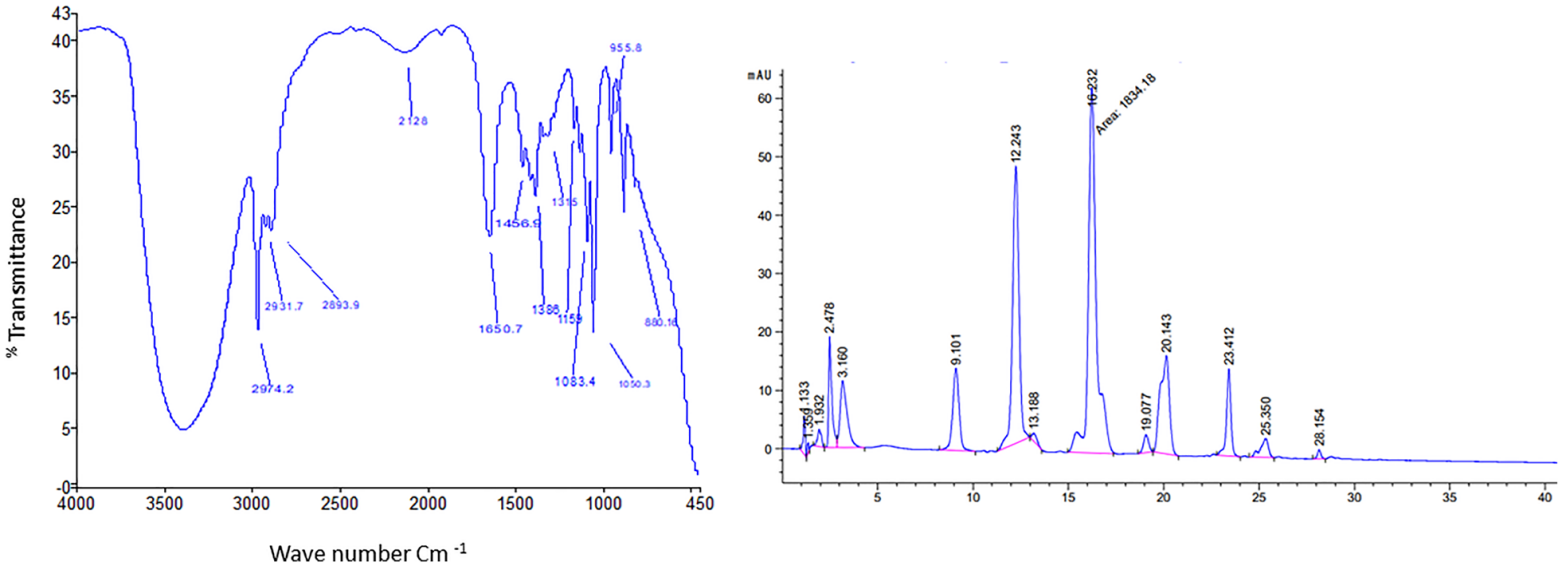
Structural analysis of *Arthrobacter agilis* NP20 carotenoids. **A** FTIR spectroscopy **B** HPLC UV spectrum

Structure determination of the extracted pigment was carried out using Fourier transform infrared (FTIR) spectroscopy, high performance liquid chromatography (HPLC) and liquid chromatography mass spectrometry (LC–MS). The FTIR analysis (Fig. 6A) showed fingerprint peak at 1650 cm^−1^corresponding to C = C alkene stretching, indicating the existence of an aliphatic group in pigment extract; in addition, a peak at 1456 cm^−1^ corresponds to CH_2_ stretching frequency. While peaks at 1388 and 1159 cm^−1^ correspond to C–H and C–OH stretching frequencies, respectively.

According to the HPLC elution conditions mentioned previously, the methanolic extract of the pigment was separated into 7 fractions (Fig. 6B). Each peak was identified based on the wavelength maxima and the MS analysis (Table 3). The detected carotenoids belong to Bacterioruberin and its derivatives.

**Table 3:** Identification of carotenoids extracted from *Arthrobacter agilis* NP20

Peak number	Retention time^a^ (min)	λ max^b^ (nm)	Cis λ max (nm)	Measured Mass (m/z)	Carotenoids identification ^c^	Formula
1	9.1	470,490,530	375,390	696 674	Bis-anhydro bacterioruberin Tetra-anhydro bacterioruberin	C_50_ H_72_O_2_ C_50_H_76_O_4_
2	12.2	470,490,540	370,390	1074 736	Bacterioruberin diglycoside Bacterioruberin	C_62_H_98_O_14_ C_50_H_76_O_4_
3	16.2	460,485,520	380,390	1069 741	Bacterioruberin diglycoside Bacterioruberin	C_62_H_98_O_14_ C_50_H_76_O_4_
4	19.07	455,490,520	-	702	Bis-anhydro bacterioruberin isomer	C_50_H_72_O_2_
5	20.1	450,480,520	360,380	901 702	Bacterioruberin mono-glycoside Bis-anhydro bacterioruberin	C_56_H_87_O_9_ C_50_H_72_O_2_
6	23.4	450,490,520	-	702	Bis-anhydro bacterioruberin isomer	C_50_H_72_O_2_
7	25.3	460,480.520	355,380	901 702	Bacterioruberin mono-glycoside Bis-anhydro bacterioruberin	C_56_H_87_O_9_ C_50_H_72_O_2_

a: HPLC retention time

b: wavelength absorption maximum of UV/visible spectra

c: carotenoids were identified based on the maximum wavelength and the corresponding mass spectra

### Antioxidant activity of *Arthrobacter agilis* NP20 carotenoid

**Fig. 7:**
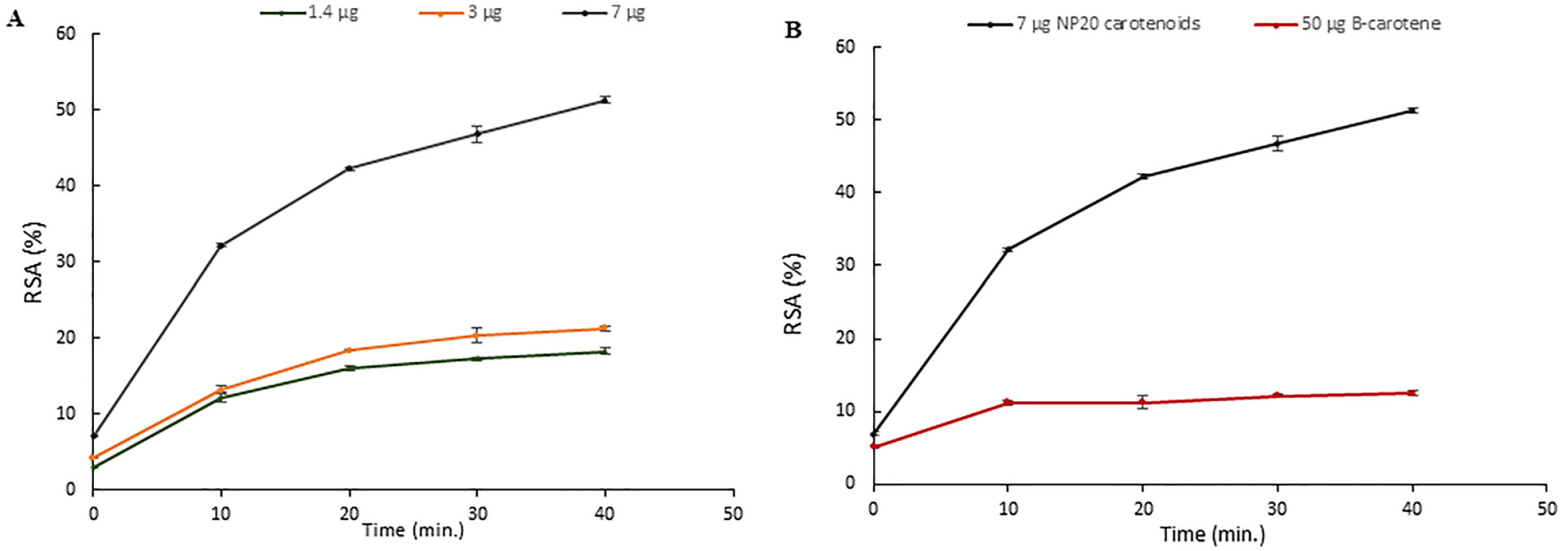
Radical scavenging efficiency of *Arthrobacter agilis* NP20 carotenoids along 40 min. **A** Radical scavenging activity of three concentrations of NP20 carotenoids, 1.4, 3 and 7 µg. **B** Radical scavenging activity of 7 µg NP 20 carotenoids compared to 50 µg β-carotene. All trials were measured at 580 nm. All trials were carried out in triplicates and values are expressed as mean ± standard error values

DPPH assay was used to assess the antioxidant activity of *Arthrobacter agilis* NP20 carotenoids. The three different concentrations of the extracted carotenoids showed a positive response with an increase in RSA% with increasing carotenoids concentration (Fig. 7A). The scavenging efficiency of NP20 carotenoids was higher than β-carotene standard. A concentration of 50 µg of β-carotene achieved a maximum RSA (12%) after 40 min, while a concentration of 7 µg of NP20 carotenoids achieved 54% (RSA %) after the same incubation time (Fig. 7B).

## Discussion

In the light of the growing demand for developing novel, risk-free, quickly biodegradable, and environmentally friendly coloring agents, microbial carotenoids have gained a great interest as an important class of bio-pigments (Mussagy et al. 2019). Carotenoids have been proven as potent antioxidant and anticancer agents, in addition, many of them including zeaxanthin and lutein, protect the eyes from cataracts and macular degeneration (Jaswir et al. 2011; Mrowicka et al. 2022). Due to their biological characteristics and possible advantages for human health, many carotenoids are currently employed as natural colorants for food, dietary supplements and nutraceuticals for pharmacological and aesthetic applications (Ashokkumar et al. 2022; Guleria et al. 2017; Gupta et al. 2022).

Bacterioruberin and its derivatives have a unique structure among the known microbial carotenoids. The structure harbors 13 conjugated double bonds distributed within a 50-carbon skeleton, that rendered it a highly potent antioxidant molecule compared to other carotenoids (Jehlička and Oren 2013; Yatsunami et al. 2014). They have a superior activity in reducing the damage of H_2_O_2_ exposure, UV and gamma irradiation (Singh and Gabani 2011; Saito et al. 1997). Owing to their antioxidant potentiality, they are found as membrane integrated carotenoids in some halophilic archaeal species, acting as photoprotection system against long lasting exposure to solar radiation. Moreover, they support the survival in hypersaline environment by controlling membrane fluidity to allow the permeability of oxygen and nutrients (Shahmohammadi et al. 1998).

In addition, bacterioruberin and its derivatives were found to be secreted in a temperature dependent manner in psychrotrophic species of Arthrobacter genus, for instance *Arthrobacter agilis* and *Arthrobacter bussei,* to enhance membrane plasticity at low temperature and protect it against freezing–thawing cycles (Fong et al. 2001b; Flegler and Lipski 2022b). Like bacterioruberin, other carotenoids have proven their role in cold adaptation, as example, *Staphylococcus xylosus* has showed a noticeable increase in staphyloxanthin production at 10 °C compared to 30 °C (Seel et al. 2020). Despite the fact that some *Arthrobacter* species synthesize bacterioruberin, the biosynthetic process in these microbes is still unknown (Flegler and Lipski 2022a).

Carotenoid glycosylation has been reported as a strategy to adapt to harsh conditions like extreme temperatures due to its role in enhancing membrane rigidity (Mohamed et al. 2005). Carotenogenic psychrotrophic species have been found to use carotenoid glycosylated derivatives to preserve the structural integrity of membranes under low temperature circumstances (Várkonyi et al. 2002). Glycosylation of carotenoids is carried out via glycosyltransferases (GTs), which is a broad enzyme family. Typically, the substituent moiety for glycosylation is a hydroxyl or carboxyl group of lipophilic substrates (Chen et al. 2021). The hydroxyl group is the most prevalent substituent moiety during carotenoid glycosylation. Bacterioruberin is glycosylated by glucosyltransferases of the GT-2 family that are unique to C50 carotenoids (Chen et al. 2021).

Glycosylation of carotenoids is not only beneficial for enhancing water solubility of carotenoids but also leads to structural diversity and many other benefits, such as enhanced bioavailability and efficacy as food and pharmaceutical supplements, improved photostability (Polyakov et al. 2009) and biological activities (e.g., antioxidant activity) (Matsushita et al. 2000). Recent studies have focused attention on generating glycosylated carotenoids via genetic engineering approaches to enhance their properties (Chen et al. 2021). In this respect, the capability of *Arthrobacter* sp. to produce glycosylated bacterioruberin is advantageous among bacterioruberin producing organisms.

In this study, C50 bacterioruberin carotenoid and its glycosylated derivatives were produced from the isolated strain NP20 of *Arhrobacter agilis* on cheese-whey-based medium as a highly nutritious, low-cost, and readily available substrate.

Cheese whey makes up 85–95% of the milk volume in dairy wastewaters, making it the most prevalent contaminant (Carvalho et al. 2013). It is considered a significant source of water pollution because of its high nitrogen content, which makes it 175 times more toxic than untreated human sewage (Smithers 2008). According to this perspective, microbial fermentation is the best method of treatment since it would lower the potential for contamination by lowering the nutritional content and turning the waste into value-added products (Zotta et al. 2020). In the introduced study, incorporating cheese-whey in the pigment production medium of the NP20 strain contributed to reducing the total nitrogen content to 75% of its value compared to the untreated whey sample.

To achieve maximum pigment yield, current studies have concentrated on understanding both nutritional and environmental factors to meet the necessary growth requirements of pigment-producing microorganisms. In this respect, statistical optimization experiments such as Response Surface Methodology (RSM) can be employed to optimize the nutritional and environmental conditions together that affect the production outcome (Cruz et al. 2020). In this study, three factors have been subjected to optimization via RSM to find the optimum response region for the maximal pigmentation in term of mg/L. The important contributing variables (MgSO_4_, whey % and inoculum %) were further investigated at three levels: − 1, 0 and + 1 to approach the pigment optimum response area with amount mg/L. The findings of surface plots revealed that raising the whey concentration and MgSO_4_ value while lowering the inoculum % (6%) resulted in higher levels of pigmentation.

Similar to the introduced study, whey-based medium was successfully used for carotenoid production from different species, for instance, β-carotene was produced from *Blakeslea trispora* using cheese whey-based medium (Roukas et al. 2015). The NP20 strain showed enhanced production of bacterioruberin with higher concentrations of MgSO4. The obtained results could be attributed to the biofunctionl role of Mg^+^ in promoting *Crt* genes expression to promote carotenoids biosynthesis. The obtained findings were in accordance with torpee et al., where higher MgSO_4_ concentrations improved carotenoid synthesis in *Rhodopseudomonas palustris* (Torpee et al. 2022). However, despite its important biofunction, the suitable concentrations of MgSO_4_ for maximum carotenoid production differ according to the genera, where higher concentration of MgSO_4_ was found inhibitory to pigment synthesis in *Monascus* sp. (Lin and Demain 1991).

It was found that the inoculum's size is a key element in carotenoid accumulation. Like NP20 strain, low inoculum size afforded higher carotenoid synthesis in *Rhodococcus opacus* PD630 compared to higher inoculum size (Thanapimmetha et al. 2017).

The *R2* value for pigment production in this design was 0.8897, indicating a strong correlation between the actual and anticipated values. The experimentally verified optimal settings from the optimization experiment were compared to the model's anticipated optimum. The estimated pigment production was 6.01 mg/L, and the polynomial model predicted a value of 5.13 mg/L. This acceptable level of % error (~ 17%) indicates that the model was validated under ideal conditions. Furthermore, pigmentation in the improved medium was 2.2 times higher than in the baseline conditions (control). This demonstrated the importance and usage of the optimization process. The RSM is a commonly accepted advanced numerical method for optimizing experimental conditions and solving analysis problems in which a response is strongly impacted by many variables for the production of many industrially important biological molecules.

A cost benefit analysis was carried out to estimate the cost effectiveness of the optimized whey-based medium in comparison to Bacto marine broth medium, the previously reported medium for bacterioruberin production medium from *Arthrobacter agilis* (Fong et al. 2001a). The formulation of whey-based medium, the prices of the added ingredients as well as that of Bacto marine broth, calculated based on the lab scale cost, are shown in (Additional file 1: Table S1). Based on the analysis, the designed whey medium was 19.6-fold less expensive than Bacto marine broth, confirming the potentiality of whey-based medium as a cost-effective medium for bacterioruberin production from *Arthrobacter agilis* NP20. In addition, the whey-based medium has supported bacterioruberin synthesis with around fivefold enhancement compared to Bacto marine broth medium at the same incubation temperature, (5.1 mg/L compared to 1.1 mg/L).

The key performance parameters and operational variables of the introduced system have been compared to bacterioruberin production from different species of Haloarchaea (Abbes et al. 2013; Montero-Lobato et al. 2018; Vega et al. 2016) (Additional file 1: Table S2). Bacterioruberin production using whey-based medium via the NP20 strain of *Arthrobacter agilis* has demonstrated advantages in terms of cost effectiveness and eco friendliness compared to the previously reported systems. Moreover, the production yield was comparable to the compared species, which would favor the introduced system for future production of bacterioruberin carotenoids and their derivatives.

Currently, the preferred methodology for carotenoid analysis is HPLC with various detection techniques, such as DAD and MS (Breemen 1997). Since many carotenoids possess a highly similar UV–Vis spectra, it’s difficult to depend on the spectral characteristics alone as a final confirmation of carotenoids identity (Breemen 1997; Crupi et al. 2010). In this context, the information obtained by MS (molecular weight and characteristic fragmentation patterns) was very helpful since it made it possible to distinguish between carotenoid components with different molecular masses.

Several diseases are thought to be reduced by the antioxidant activity of carotenoids, and some of them, like lycopene and β-carotene, have well-known antioxidant qualities (Suleman et al. 2019). Carotenoids can be used as colorants or functional components in the food, cosmetic, and pharmaceutical industries thanks to their antioxidant action (Agarwal et al. 2023). The addition of carotenoids to foods may have significant positive effects on health, and their usage as antioxidants in the cosmetic industry is growing even more (Anunciato and Rocha Filho 2012). The high potentiality of bacterioruberin carotenoids in scavenging RSA is attributed to its long conjugated double bond system in comparison to other carotenoids. A concentration of 7 µg of NP20 carotenoids achieved (RSA%) of 54% while 50 µg of β-carotene achieved 12% only. The extract's enhanced effects can be attributed to the presence of several components that work in concert with one another. This outcome supports the benefit of employing natural blends rather than standalone antioxidants (Squillaci et al. 2017). Whole extracts rather than individual BR molecules are more closely related to the prospective applications of NP20 carotenoids.

## Conclusion

Cheese whey-based medium has been proven as a potent nutritious substrate for producing bacterioruberin carotenoid and its unique glycosylated derivatives from *Arthrobacter agilis* NP20. The cost effectiveness of the newly developed medium creates a solid opportunity for bacterioruberin manufacturing on a wide scale. The low production cost of bacterioruberin carotenoid on whey-based media would facilitate the use of this rare carotenoid in a variety of sectors, including the food and cosmetics industries.

### Supplementary Information

**Additional file 1: Table S1.** Cost analysis of Bacto marine broth and whey-based medium for bacterioruberin production. **Table S2.** Key performance parameters indicators and operational variables of bacterioruberin pigment production from different microorganisms. **Figure S1. **Actual by predicted plot for the measured response Y (Pigment conc. (mg/L)).

## Data Availability

All data generated or analyzed during this study are included in this published article.
