# Supplementary material for: Sustainable production of bacterioruberin carotenoid and its derivatives from Arthrobacter agilis NP20 on whey-based medium: optimization and product characterization
Source: Bioresour Bioprocess. 2023 Jul 24;10(1):46. doi: 10.1186/s40643-023-00662-3 (PMC10991996; doi:10.1186/s40643-023-00662-3)
Supplement: Supplementary file 1 — Additional file 1: Table S1. Cost analysis of Bacto marine broth and whey-based medium for bacterioruberin production. Table S2. Key performance parameters indicators and operational variables of bacterioruberin pigment production from different microorganisms. Figure S1. Actual by predicted plot for the measured response Y (Pigment conc. (mg/L)). [file 40643_2023_662_MOESM1_ESM.doc]

| **Medium** | **Ingredients** | **Amount added per Liter** | **Cost per Liter medium**  **($/L)** | **Total medium cost**  **($/L)** |
| --- | --- | --- | --- | --- |
| **Bacto marine broth** | As purchased | 37.4 g | 31 | 31 |
| **Whey-based medium** | Yeast extract | 5g | 1.5 | 1.58 |
|  | MgSO_4_ | 0.47g | 0.08 |  |
|  | Cheese whey | 0.98L | - |  |

Additional file 1: Table S1: Cost analysis of Bacto marine broth and whey-based medium for bacterioruberin production.

| **Performance**  **and parameters**  **Indicators**  **Producer strains** | **Production Medium** | **Medium cost**  **($/L)** | **Stress manner**  **for pigment synthesis** | **Temp.** | **pH** | **Agitation rate**  **(rpm)** | **NaCl conc.**  **(g/L)** | **Production Time**  **(Days)** | **Pigment yield**  **(mg/L)** | **Environmental impact** | **References** |
| --- | --- | --- | --- | --- | --- | --- | --- | --- | --- | --- | --- |
| *Arthrobacter agilis* NP20 | Cheese whey-based medium | 1.58 | Temperature | 20 ^o^C | 7.0 | 150 | - | 3 | 5.6 | Treated cheese whey effluent | Current study |
| *Arthroacter agilis* MB813 | Bacto marine broth | 31 | Temperature | 20 ^o^C | 7.0 | 150 | - | 3 | 1.1 | - |  |
| *Halobacterium halobium* | DSC-97 broth | 26 | Salinity | 37 ^o^C |  | 240 | 250 | 7 | 7.63 | Producing extreme salty effluents | (19) |
| *Haloferax mediterranei*  ATCC 33500T | Archaea specific medium | 14 | Salinity | 37 ^o^C | 8.9 | 150 | 156 | 7 | 3.34 | Producing extreme salty effluents | (55) |
| *Halorubrum sp. SH1* | Archaea specific medium | 16 | Salinity | 37 ^o^C | 7.0 | 550 | 250 | 8 | 20 | Producing extreme salty effluents | (56) |

Additional file 1: Table S2: Key performance parameters indicators and operational variables of bacterioruberin pigment production from different microorganisms

Additional file 1: Figure S1: Actual by predicted plot for the measured response Y (Pigment conc. (mg/L)).
